# Spectroscopic strategies for quantitation of varenicline in pharmaceutical preparations and content uniformity testing

**DOI:** 10.1098/rsos.220628

**Published:** 2022-09-28

**Authors:** Fawzia Ibrahim, Rasha Aboshabana, Heba Elmansi

**Affiliations:** Pharmaceutical Analytical Chemistry Department, Faculty of Pharmacy, Mansoura University, 35516 Mansoura, Egypt

**Keywords:** erythrosine B, varenicline, fluorescence, quenching, spectrophotometry

## Abstract

Herein, two new facile methods were examined for varenicline determination using erythrosine. The latter is a food additive that has been recently investigated as a fluorescent dye for the determination of drugs. In the first method, the fluorescence of erythrosine B was quenched quantitatively by increasing the concentration of varenicline through ion-pair complex formation. This linear response was a basis for the spectrofluorimetric method used for varenicline quantitation in pure and dosage forms. The quenching is correlated with the concentration linearly over the range of 0.4–4.0 µg ml^−1^ at 550 nm after excitation at 528 nm with a correlation coefficient of 0.9993. Different parameters were investigated to reach the optimal conditions with the highest sensitivity and repeatability. The second method is depending on measuring the formed complex by spectrophotometry at 550 nm over the range of 1.0–10.0 µg ml^−1^ with an excellent correlation coefficient of 0.9999. The suggested methods were validated consistently with ICH guidelines, with acceptable results. The procedures were used to test the uniformity of content of Champix tablets. By comparing with the previous spectroscopic method, there was no significant difference as revealed from the calculated Student *t*-test and variance ratio *F*-test values.

## 1. Introduction

**Figure 1.**
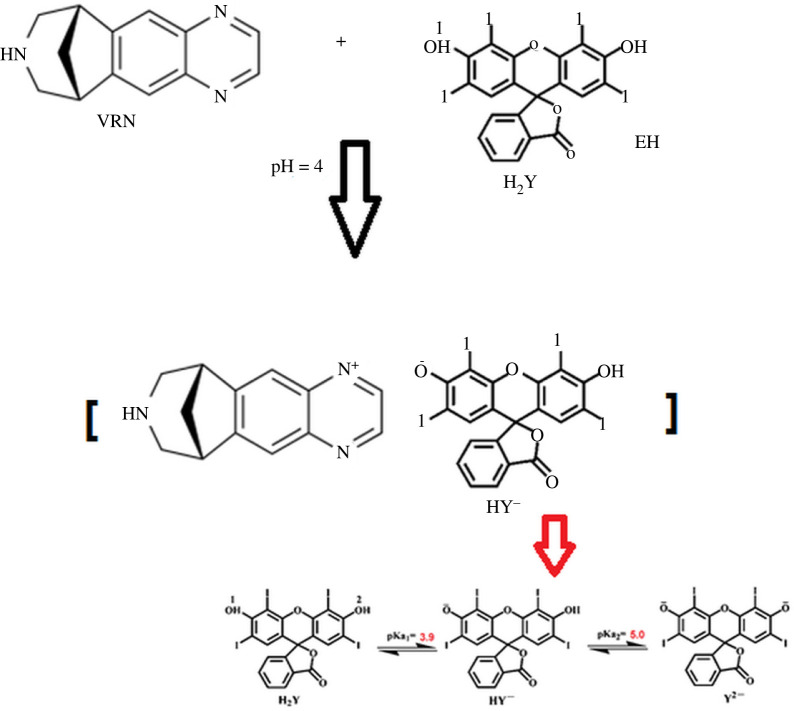
The proposed reaction pathway between VRN and EB at pH 4 indicating the dissociation of EB at different pH values.

Varenicline (figure 1), VRN, is a relatively recent drug used for helping adults to stop smoking. It is chemically: 7, 8, 9, 10-Tetrahydro-6, 10-methano-6H pyrazino [2, 3-h] benzazepine (2R, 3R)-2,3dihydroxybutanedioate.^1^ Varenicline is centrally acting as a highly selective partial agonist for the nicotinic acetylcholine receptor as nicotine replacement therapy [1]. This therapy aims to reduce smoking tobacco without the dangerous effects of cigarettes [2,3]. The usual oral dosage in adults is 1 mg twice daily for 12 weeks. Different methods have been designated for the quantitation of VRN in its bulk and tablets using spectrofluorimetry [4,5], spectrophotometry [6], HPLC [4,7–11], capillary electrophoresis [12] and LC/MS/MS [13] in addition to pharmacokinetic studies [13,14]. Additionally, electrochemical methods have been reported [14].

Erythrosine B (EB) (figure 1) is 3′,6′-dihydroxy-2′,4′,5′,7'-tetraiodospiro[2-benzofuran-3,9'-xanthene]-1-one (see footnote 1). It is a tetraiodo-fluorescein used as a red colouring agent for food, as a disclosure of dental plaque, and as a stain of some cell types. Erythrosine B is also used as a spectrofluorimetric reagent for determining some drugs including imipramine [15], fluoroquinolones [16], flubendazole, mebendazole [17], tamoxifen and clomiphene [18].

As revealed from the literature, few methods determine VRN using spectrofluorimetry or spectrophotometry. In addition, some of the reported methods depended on derivatization reactions, used heating and hazardous solvents including acetonitrile [7], or used a gradient elution system. HPLC procedures usually require a high quantity of organic solvents, multiple sample pretreatment procedures, and complicated and expensive devices and detectors. A previous method suggested NBD-Cl for VRN determination, but it involved heating steps and using HCl [5]. Therefore, in the current study, we aim to develop facile, accurate, reproducible, cheap and time-saving spectrofluorometric and spectrophotometric methods for the determination of VRN using the self-fluorescent dye erythrosine B. The utility of this reagent in food makes the method environmentally friendly and no hazards are expected from its use in the analysis. Hence, our proposed methods are considered fast, convenient and eco-friendly, which makes them superior to previous methods.

## 2. Experimental set-up

### 2.1. Instrumentation

For performing the spectrofluorimetric measurements, Cary Eclipse fluorescence spectrophotometer from Agilent Technologies, with a xenon flash lamp, was used. It was adjusted at voltage of 670 V. The quenching of the fluorescence of erythrosine B was measured at 550 nm after excitation at 528 nm, with smoothing factor 20.

For the spectrophotometric and the comparison methods, 6850 double beam spectrophotometer was used (Jenway).

### 2.2. Materials, solvents and reagents

Methanol, ethanol and acetonitrile were HPLC grade. Double-distilled water was used for washing, preparation and dilutions when needed.

Erythrosine B was purchased from Acros-organics, Cole-Parmer 625 East Bunker Ct Vernon Hills, IL 60061 USA. Certified (25 g) CAS 16423-68-0. It was prepared as 1.7 × 10^−4^ and 5 × 10^−4^ M solutions in distilled water for the spectrofluorimetric and spectrophotometric methods, respectively.

Britton Robinson (BR) buffer was prepared by using phosphoric acid, boric acid and acetic acid, all of 0.04 M concentration, and the pH was adjusted by 0.2 M sodium hydroxide. The chemicals used were purchased from Merck KGaA, Darmstadt, Germany. The purity of phosphoric acid, boric acid, acetic acid and sodium hydroxide were 99.99%, 99.5%, 99% and 98%, respectively, as certified.

Varenicline pure powder (99.96% as labelled) was purchased from Weihua Pharma Co. Ltd (Zhejiang, China). The purity was also assessed by the comparison method [6].

Champix® tablets, labelled to contain 0.5 mg and 1.0 mg VRN (as tartrate) per tablet, were obtained from the local market, manufactured by Pfizer Manufacturing Deutschland GmbH, Germany and Marketing Authorization Holder: Pfizer Limited, UK.

### 2.3. Preparing of the calibration solutions

Stock containing 100.0 µg ml^−1^ of VRN was prepared by dissolution of 10.0 mg in 90 ml distilled water and adjusting the final volume to the mark in a 100 ml volumetric flask. The solution is freshly prepared, and suitable volumes of distilled water were added when dilutions were needed to get the working solutions.

### 2.4. Optimization of the experimental parameters

Different variables including pH, buffer volume, reagent volume and diluting solvents were studied using 5.0 µg ml^−1^ VRN for the spectrophotometric method. Each parameter was optimized separately while keeping others constant.

### 2.5. Calibration curves construction

#### 2.5.1. The spectrofluorimetric method

A sequence of 10 ml volumetric flasks was prepared. Aliquots were transferred to it from the stock solution of VRN to reach the final concentration range of (0.4–4.0 µg ml^−1^). One millilitre of BR buffer of pH 4 and 0.4 ml of 1.7 × 10^−4^ M erythrosine B were added. Volumes were then completed to the mark with distilled water. The response was measured using the spectrofluorimeter at 550 nm after excitation at 528 nm. A calibration curve was constructed, and the corresponding regression equation was derived.

#### 2.5.2. The spectrophotometric method

Different aliquots of VRN were transferred to another set of 10 ml flasks to reach the final concentration range of 1.0–10.0 µg ml^−1^. One millilitre of BR buffer (pH 4) and 3.0 ml of 5 × 10^−4^ M erythrosine B were added. Volumes were completed with distilled water; then absorbance was recorded using the spectrophotometer at 550 nm against reagent blank. Subsequently, the calibration graph was constructed and the corresponding regression equation was then derived.

### 2.6. Analysis of Champix® tablets and assessment of content uniformity

Ten tablets from Champix^®^ were ground thoroughly, and an amount of the powder equivalent to 100.0 mg of VRN was placed in a 100 ml volumetric flask. Subsequently, 60 ml of water was added, the flask was sonicated for 40 min, then completed with water to the mark. The solution was subjected to filtration discarding the first few millilitres. Accurate volume of the clear solution was quantitatively transferred to another 100 ml volumetric flask and completed with distilled water to yield 100.0 µg ml^−1^ solution. Measured volumes from the last solution were transferred to accomplish the procedures described in both spectrofluorimetric and spectrophotometric methods. Tablet content was computed by referring to the regression equations.

For assessment of content uniformity, 10 tablets were individually weighed and transferred separately to a 100 ml volumetric flask. The procedure described for the analysis of VRN tablets was followed. The uniformity of their contents was evaluated by applying the official USP guidelines [19] (Chapter 905: Uniformity of dosage units).

## 3. Results and discussion

**Figure 2.**
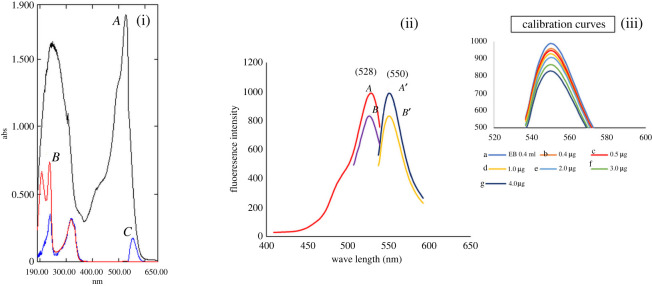
(i) UV spectra of: *A* 3.0 ml of EB (5 × 10^−4^ M) *B*: 10 µg ml^−1^ of VRN in water. *C*: reaction product of 1.0 µg ml^−1^ of VRN with of EB (5 × 10^−4^ M). (ii) Fluorescence spectra of: (*A, A'*) excitation and emission spectra of 0.4 ml of EB (1.7 × 10^−4^ M) and (*B, B'*) excitation and emission spectra of VRN-EB complex. (iii) Emission spectra of: Reaction product of different concentration of VRN in BRP (pH 4.0) with 0.4 ml of EB where a: blank and b–g the reaction product of EB with VRN at concentrations from 0.4 to 4.0 µg ml^−1.^

Erythrosine B shows a strong fluorescence emission at 550 nm after excitation at 528 nm. The cited drug was found to react with erythrosine B in BR buffer at pH = 4 to form an ion-pair complex which could be quantitated using both techniques without prior extraction and heating steps (figure 1). The complex is measured by spectrophotometry at 550 nm. Also, subsequent decrease in the fluorescence intensity of the reagent occurred upon the formation of VRN-EB complex (figure 2), and this decrease is directly related to the concentration of VRN. Critical variables that may affect the reaction were investigated to get the optimum conditions.

### 3.1. Effect of pH and buffer volume

**Figure 3.**
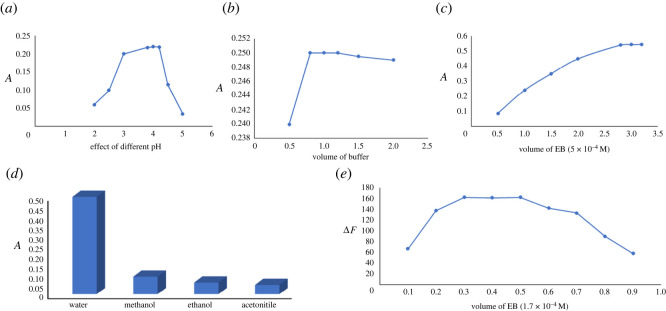
(*a*) Effect of pH of BRB on absorbance (studied concentration is 5.0 µg ml^−1^). (*b*) Effect of volume of BRB on absorbance (studied concentration is 5.0 µg ml^−1^). (*c*) Effect of volume of EB (5 × 10^−4^) on the absorbance (studied concentration is 5.0 µg ml^−1^). (*d*) Effect of different diluting solvents on the absorbance (studied concentration is 5.0 µg ml^−1^). (*e*) Effect of volume of EB (1.7 × 10^−4^) on the fluorescence quenching (studied concentration is 4.0 µg ml^−1^).

As illustrated in figure 1, the acidic dye EB requires acidic pH to form the complex with VRN. Different pH values from 2.0 to 5.0 were studied using BR buffer. The highest response in both methods was achieved using pH 4. Volumes were varied from 0.5 to 2.0 ml buffer, and fluorescence quenching or absorbance values were increased with volumes of BR buffer up to 0.8 ml. After this point, increasing the buffer volume has insignificant effect on the results. Thus, 1.0 ml of buffer solution was optimum for this reaction (figure 3). Decreasing the volume of buffer may be insufficient to keep the pH, and larger volumes increase the ionic strength of the solution, so that the positive component of the buffer competes with VRN cations for coupling with the anionic dye. This effect would hinder the complex formation [20].

### 3.2. Effect of different diluting solvents

Different diluting solvents were assessed to reach the highest response for this reaction, including ethanol, methanol, water and acetonitrile. As shown in figure 3, it was concluded that water gives the greatest values, and hence it was the optimum solvent. The advantages of using water include availability, cost and eco-friendly properties which make this method superior to previous methods.

### 3.3. Effect of volume of erythrosine B

The concentration and volume of the reagent were optimized for the two methods separately. The optimum concentrations for EB were 1.7 × 10^−4^ and 5 × 10^−4^ M solutions for the spectrofluorimetric and spectrophotometric methods. It was found that 0.4 ml of EB resulted in high quenching values. Meanwhile, 3.0 ml was suitable volume in the spectrophotometric method using the specified concentration.

### 3.4. Effect of reaction time

The time of the reaction was investigated by measuring the quenching instantly and after 5, 10, 15, 20 and 30 min. It was found that the time increase did not affect the fluorescence of the formed complex, so the product was measured instantaneously.

### 3.5. Stoichiometry of the reaction

**Figure 4.**
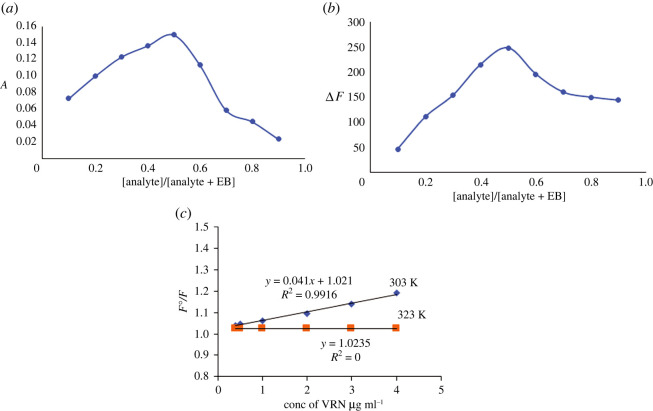
(*a*,*b*) Continuous variation (Job's) plots for determination of stoichiometry of reaction of VRN and EB at concentrations of 5 × 10^−4^ and 1.7 × 10^−4^ M, respectively. (*c*) Stern–Volmer plots for fluorescence quenching at 303 and 323 K, using 1.7 × 10^−4^ M EB at pH 4.

**Figure 5.**
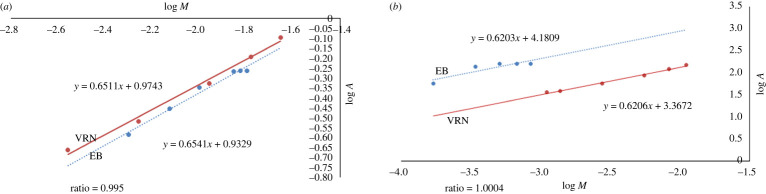
Limiting logarithmic plots for determination of molar ratio of reaction: (*a*) log *A* versus log VRN at fixed concentration of EB; (*b*) log *A* versus log EB at fixed concentration of VRN.

To assess the stoichiometry of the reaction, Job's method of continuous variation was followed. Solutions of equal molarity of both the VRN and EB were mixed so that the total complementary concentrations are constant. The fluorescence intensity for the VRN-EB mixture was subtracted from the fluorescence intensity of the solutions with the same quantity of EB only. Job's plot was constructed. Limiting logarithmic method was also applied by using equimolar concentration of VRN and EB. It was concluded previously that the hydroxyl group tends to separate easier than the carboxylic owing to the two iodine particles next to the hydroxyl gather [16]. VRN has an amino group protonated within the acidic medium to form a complex with the reagent in 1 : 1 ratio as confirmed by Job's plot and limiting logarithmic method (figures 4 and 5).

The quenching of erythrosine B fluorescence may have occurred because of different causes including energy transfer, collisional quenching and excited state reactions. Therefore, Stern–Volmer plot was constructed at 303 and 323 K (figure 4) and resulted in decreasing slopes of the plots with increasing temperature, which is indicative for static quenching [17].

### 3.6. Validation parameters

The developed procedures were validated concerning International Conference of Harmonization (ICH) guidelines [21].

Following the suggested experimental criteria, a linear relationship was obtained in the range of 0.4–4.0 µg ml^−1^ in the spectrofluorimetric method and the range of 1.0–10.0 µg ml^−1^ in the spectrophotometric method. Different analytical parameters obtained are listed in table 1.

**Table 1.** Validation data for the proposed methods.

parameters	spectrofluorimetric method	spectrophotometric method
wavelength	*λ*_ex_ = 528, *λ*_em_ = 550 nm	*λ*_max_ = 550 nm
calibration range (µg ml^−1^)	0.40–4.00	1.00–10.00
intercept (*a*)	21.34	0.14
slope (*b*)	33.87	0.083
correlation coefficient (*r*)	0.9994	0.9999
s.d. of residuals (*S_y/x_*)	1.91	1.90 × 10^−3^
s.d. of intercept (*S_a_*)	1.32	1.56 × 10^−3^
s.d. of slope (*S_b_*)	0.59	5.10 × 10^−4^
percentage relative standard deviation, % RSD	1.83	0.37
percentage relative error, *%* error	0.75	0.15
limit of detection, LOD (µg ml^−1^)	0.13	0.06
limit of quantitation, LOQ (µg ml^−1^)	0.39	0.19
Sandell's sensitivity (µg/cm^2^)		0.01

Limits of detections (LOD) and quantitation (LOQ) were computed by the mathematical formulae illustrated by ICH guidelines [21]. These equations are LOD = 3.3 S_a_/b, LOQ = 10 S_a_/b, where *S_a_* is the standard deviation of the intercept of the regression line and *b* is the slope. The lowest detected concentrations (LOD) were found to be 0.13 and 0.06 µg ml^−1^ for the spectrofluorimetric and spectrophotometric methods respectively. LOQ were 0.39 and 0.19 µg ml^−1^, respectively, which reveals the adequate sensitivity of the proposed methodologies. Sandal's sensitivity is obtained for the spectrophotometric method by calculating the concentration at 0.001 absorbance unit.

To confirm the accuracy, the data obtained from both methods were compared with a comparison spectroscopic method [6]. The method is depending on UV–Vis spectrophotometric determination for VRN at 319 nm in 0.01 M phosphate buffer at pH = 7. Statistical evaluation of the results indicates no significant differences concerning the proposed methods' results and the previous method as revealed by Student's *t*-test and the variance ratio test (*F*-test) [22] (table 2).

**Table 2.** Application of the proposed method for the assessment of VRN in pure forms. Each result is the average of three separate determinations.

method	amount taken (µg ml^−1^)	amount found (µg ml^−1^)	% found	comparison method [6] % found
spectrofluorimetric method	0.400	0.401	100.25	99.40
	0.500	0.509	101.80	101.35
	1.000	1.02	102.00	102.1
	2.000	1.953	97.65	
	3.000	2.952	98.40	
	4.000	4.055	101.38	
$\bar{x}\pm s.d.$			100.25 ± 1.84	100.95 ± 0.48
*t*			0.59 (2.77)*	
*F*			14.69 (19.25)*	
spectrophotometric method	1.000	0.996	99.60	99.40
	2.000	1.995	99.75	101.35
	4.000	4.004	100.10	102.1
	6.000	6.035	100.58	
	8.000	7.969	99.61	
	10.000	10.001	100.01	
$\bar{x}\pm s.d.$			100.01 ± 0.37	100.95 ± 0.48
*t*			2.24 (2.44)*	
*F*			1.68 (5.79)*	

*The tabulated *t* and *F* values were obtained at *p* = 0.05 [18].

The precision of the proposed methods was investigated at two levels, intra- and inter-day precision. Three different concentrations within the linear ranges 1.0, 2.0, 3.0 µg ml^−1^ and 4.0, 6.0, 8.0 µg ml^−1^ for the spectrofluorimetric and spectrophotometric methods, respectively, were examined three times within one day and at three succeeding days (table 3). The values of the percentage relative standard deviation (% RSD) and percentage error were low, proving the adequate precision of the methods [22].

**Table 3.** Precision data for the assessment of VRN by the proposed methods.

amount taken (mg/ml)	% found	% RSD	% error
spectrofluorimetric method			
Intra-day 1.0	100.9 ± 1.80	1.81	1.04
2.0	98.89 ± 1.13	1.14	0.66
3.0	99.39 ± 0.87	0.88	0.51
Inter-day 1.0	100.60 ± 1.20	1.21	0.69
2.0	99.19 ± 1.21	1.22	0.70
3.0	100.05 ± 1.65	1.64	0.95
spectrophotometric method			
Intra-day 4.0	100.26 ± 0.27	0.27	0.15
6.0	99.78 ± 0.77	0.77	0.45
8.0	98.79 ± 1.19	1.19	0.69
Inter-day 4.0	99.12 ± 0.76	0.76	0.44
6.0	99.46 ± 0.64	0.64	0.37
8.0	99.08 ± 1.18	1.18	0.69

The robustness of the developed methods was investigated by monitoring the changes in the analytical response upon minor changes in the experimental conditions. This includes pH (4.0 ± 0.2), buffer volume (1.0 ± 0.2 ml) EB volume (0.4 ± 0.1 ml) or (3.0 ± 0.5 ml) for the spectrofluorimetric or the spectrophotometric methods. These parameters were changed giving acceptable per cent recovery and standard deviation values. So, there was no significant effect on the fluorescence quenching or the absorbance readings, proving the robustness of the proposed approaches.

### 3.7. Application to Champix® and content uniformity testing

Both spectroscopic methods were efficiently applicable for the assay of VRN in its tablets and to assess the uniformity of these tablets. Different ingredients of tablets did not interfere with the proposed methods as indicated by good percentage recovery. These ingredients include calcium hydrogen phosphate anhydrous, microcrystalline cellulose, croscarmellose sodium, silica and colloidal anhydrous magnesium stearate. The results were also compared with the previous UV method concerning accuracy and precision and they were satisfactory (table 4).

**Table 4.** Application of the proposed methods for determination of varenicline in tablets. Each result is the average of three separate determinations.

spectrofluorimetric method				
sample	amount taken (µg ml^−1^)	amount found (µg ml^−1^)	% found	comparison method [6] % found
Champix 1 mg tab. (1 mg VRN/tablet)	1.0	1.01	100.6	99.00
	2.0	1.98	98.8	101.00
	3.0	3.00	100.1	100.5
$\bar{x}\pm s.d.$			99.83 ± 0.92	100.17 ± 1.04
*T*			0.44 (3.18)*	
*F*			1.28 (5.79)*	
Champix 0.5 mg tab. (0.5 mg VRN/tablet)	1.0	1.01	101.0	99.89
	2.0	1.98	98.8	101.00
	3.0	3.06	102.0	100.78
$\bar{x}\pm s.d.$			100.27 ± 1.27	100.56 ± 0.58
*t*			0.44 (4.33)*	
*F*			4.79 (19.30)*	
spectrophotometric method				
sample	amount taken (µg ml^−1^)	% found	% found	comparison method [6] % found
Champix 1 mg tab. (1 mg VRN/tablet)	4.0	4.02	100.6	99.00
	8.0	7.95	99.4	101.00
	10.0	9.99	99.9	100.5
$\bar{x}\pm s.d.$			99.97 ± 0.60	100.17 ± 1.04
*t*			0.33 (4.33)*	
*F*			3.0 (5.79)*	
Champix 0.5 mg tab. (0.5 mg VRN/tablet)	4.0	4.02	100.6	99.89
	8.0	8.00	100	101.00
	10.0	10.01	100.1	100.78
$\bar{x}\pm s.d.$			100.23 ± 0.32	100.56 ± 0.58
*t*			0.82 (4.33)*	
*F*			3.29 (5.79)*	

*The tabulated *t* and *F* values are obtained at *p* = 0.05 [18].

Additionally, the proposed methods are convenient for investigating the content uniformity testing of the tablets. The test was adopted as stated by the USP procedures [19]. The acceptance value (AV) was computed to get a value lower than the maximum allowed AV (L1). The results in table 5 confirmed excellent drug uniformity.

**Table 5.** Application of the proposed methods for assessment of content uniformity of VRN tablets. The bold values refer to acceptance value (AV) and max. allowed AV (L1).

parameter	tablet no.	percentage of the label claim			
		spectrophotometric method		spectrofluorimetric method	
		Champix 0.5 mg	Champix 1.0 mg	Champix 0.5 mg	Champix 1.0 mg
data	1	99.60	97.88	102.10	100.25
	2	99.75	101.80	101.12	101.8
	3	100.1	100.60	100.99	102
	4	100.58	98.90	99.56	97.65
	5	99.61	97.98	99.76	98.4
	6	100.01	100.55	100.45	101.38
	7	100.26	100.90	101.66	99.76
	8	99.46	100.1	99.56	100.50
	9	97.90	100.23	99.70	102.10
	10	99.12	97.76	101.30	98.80
mean ($X{;}^{-}$)		99.64	99.67	100.62	100.26
s.d.		0.74	1.43	0.94	1.59
% RSD		0.745	1.43	0.936	1.58
% error		0.235	0.453	0.298	0.50
acceptance value (AV) [20]		**1.77**	**3.43**	**2.26**	**3.82**
max. allowed AV (L1) [20]		**15.00**	**15.00**	**15.00**	**15.00**

### 3.8. Consideration of the green property

**Figure 6.**
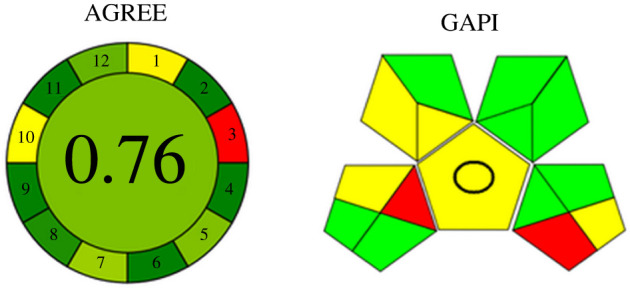
Results of AGREE and GAPI analysis for the proposed spectroscopic methods.

To emphasize the importance of the eco-friendly analysis of pharmaceutical drugs, we investigated the proposed methods using the Analytical GREEnness metric approach (AGREE) and Green Assessment Profile Index (GAPI) [23,24]. For AGREE, it is a tool for assessing the hazards accompanying an analytical procedure [23]. This approach investigates the 12 principles of green analytical chemistry [25]. It results in a numerical overall value that assists the view of the whole methodology. As we get closer to 1, it is considered a greener methodology. From figure 6, it is shown that our methods scored 0.76 in the AGREE analysis, so they are regarded as eco-friendly approaches. Regarding GAPI as a semiquantitative tool for estimating greenness; figure 6 indicates that most parameters are coloured green, a few are coloured yellow and only two parameters are red, which referred to off-line sampling and the absence of waste treatment in the proposed procedures.

## 4. Conclusion

Erythrosine B was investigated as a facile probe for VRN selective estimation. The developed methods were based on developing an ion-pair complex between this dye and VRN. The linear ranges were 0.4–4.0 and 1.0–10.0 µg ml^−1^ for the spectrofluorimetric and spectrophotometric methods, respectively. Different advantages of the proposed methods have been revealed, including facility, time and cost-effectiveness in addition to eco-friendly properties. The methods were efficiently applied to VRN analysis in tablets without any interference from excipients as revealed by high percentage recovery values. Additional examination of the content uniformity of tablets was performed. The results obtained in the present study provide facile alternatives for the quality control tests of commercial tablets of VRN.

## Data Availability

Data available from the Dryad Digital Repository: https://doi.org/10.5061/dryad.95x69p8nj [26].

## Footnotes

1 https://iupac.org/ (accessed 1 October 2021).
